# Follicular Fluid Oocyte/Cumulus-Free DNA Concentrations as a Potential Biomolecular Marker of Embryo Quality and IVF Outcome

**DOI:** 10.1155/2014/289306

**Published:** 2014-06-15

**Authors:** M. Dimopoulou, G. Anifandis, C. I. Messini, K. Dafopoulos, S. Kouris, S. Sotiriou, M. Satra, N. Vamvakopoulos, I. E. Messinis

**Affiliations:** ^1^Department of Obstetrics and Gynecology, School of Health Sciences, Faculty of Medicine, University of Thessaly, 41222 Larissa, Greece; ^2^Department of Molecular Biology, School of Health Sciences, Faculty of Medicine, University of Thessaly, 41222 Larissa, Greece

## Abstract

The present prospective study examined the follicular fluid oocyte/cumulus-free DNA concentrations (ff o/c-free DNA) during ovarian stimulation and the possible association between ff o/c-free DNA and embryological results such as embryo quality and pregnancy rate. Eighty-three women undergoing IV/ICSI-ET treatments were prospectively included in this study. ff o/c-free DNA was determined by conventional quantitative real time PCR-Sybr green detection approach. The 83 ff samples were categorized in two groups: group 1 (*n* = 62) with cumulus oocytes complexes (CoCs) ≥2 and group 2 (*n* = 21) with CoCs = 1. Group 1 revealed significant higher embryo quality in terms of mean score of embryo transfer (MSET), but lower ff o/c-free DNA concentrations compared to group 2. The two groups showed comparable pregnancy rates (positive hCG and clinical pregnancy). The higher the ff o/c-free DNA concentration, the lower the number of produced oocytes. ff o/c-free DNA did not seem to have any direct role in the IVF outcome. Further research is required to clarify whether ff o/c-free DNA is a biomolecular marker of embryo quality and IVF outcome.

## 1. Introduction

Cell-free DNA (cf-DNA) is fragments of DNA that have been found in body fluids of both healthy individuals and patients. It is released from the cell nucleus mainly during apoptotic processes [1]. Recent reports have emphasized the importance of studying the presence of cf-DNA and the possible association with various diseases. For that reason it has been reported that increased cf-DNA concentrations have been measured in cancer [2–4] and preeclampsia [5]. Elevated fetal cf-DNA is associated with preeclampsia in pregnancy, since fetal cf-DNA has also been detected in serum of pregnant women [6].

So far, research has focused on which factors are strongly related to IVF success or failure. The reports concerning one putative predictive factor of IVF outcome, the serum cf-DNA, are scarce. Serum cf-DNA has been proposed as a marker of semen quality, since it has been associated with important sperm parameters linked to normal sperm function [7]. It has been reported that there is no relationship between serum cf-DNA concentrations and IVF outcome in patients undergoing IVF-ET treatments [8], while a more recent report indicated that increased serum cell-free DNA is associated with low pregnancy rate in women undergoing IVF-ET programs [9]. Methods used for the analysis of serum cf-DNA are real-time polymerase chain reaction (PCR) [3, 10] and quantitative PCR [2], while relative recently a new simple and highly sensitive method known as the SYBR gold fluorescent-stained direct assay has been developed [11].

The present study examined for the first time oocytes/cumulus-free DNA (o/c-free DNA) in the follicular fluid (ff), the ambient environment of the oocytes, in women undergoing IVF/ICSI-ET treatments with the aim to investigate the role of this factor as a potential biomolecular marker affecting embryo quality and subsequent IVF outcome.

## 2. Materials and Methods

Eighty-three ff samples were obtained from 83 women undergoing 83 consecutive IVF/ICSI-ET treatments in our Assisted Conception Unit. All women were prospectively investigated after informed consent and approval of Institutional Review Board.

All women were allocated to two groups. In group 1 the number of collected CoCs was more than or equal to two and group 2 the number of produced CoCs was equal to one. The biological rationale for this categorization, after performing the same ovarian hyperstimulation protocol for all women, was to investigate whether the ff o/c-free DNA, which is produced by the cumulus cells and the oocytes, differed between women who produced 1 oocyte from women who produced more than 1 oocyte. All 83 studied women reached the embryo transfer process either two or three days postinsemination. Serum *β*-hCG levels were assessed 12 to 13 days postembryo transfer and levels greater than 15 IU/mL were considered positive. Clinical pregnancy was defined when an intrauterine sac with heartbeat was seen by ultrasound 4 to 5 weeks post-hCG.

### 2.1. Exclusion Criteria

Poor responders were excluded from the study; at first, there is a difference in ovarian stimulation between poor and normal responding women (in contrast to the present study all women received the same ovarian stimulation protocol) and, secondly, poor responders produce low number of oocytes (≤4) in contrast to normal responders and therefore it is opposite from our biological rationale for the categorization used above. Women older than 42 years, couples who did not finally reach the embryo transfer procedure for various reasons (e.g., fertilization failure and embryo developmental arrest), or couples with donor semen samples were excluded from the study.

### 2.2. Ovarian Stimulation

All women received the same ovarian stimulation protocol. Briefly, ovarian stimulation was performed by administration of recombinant FSH (Puregon, N.V. Organon, or Gonal-F Serono) in a short GnRH agonist protocol. Treatment started on day 2 of the cycle with a FSH dose of 2–6 ampoules (75 IU each ampoule) depending on the age of every woman. This dose was given for 4 days and then it was modified according to the ovarian response. When at least two follicles reached a diameter of 17 mm, 5000–10000 IU of human chorionic gonadotropin (Pregnyl, N.V. Organon) was administered and, 34–36 hours later, ovum pickup (OPU) was performed under light sedation.

### 2.3. Fertilization and Embryo Score

Fertilization was evaluated approximately 18–20 hours after insemination by the presence of two pronuclei and extrusion of the second polar body. Embryos were evaluated either on day 2 or day 3. Embryo score was performed as described previously [12]. Briefly, morphological grade was performed including the number of blastomeres (2–8), the amount of the fragmentation (1–4, with the fourth scale representing no fragmentation), and the regularity of the blastomeres (1-2, with the second scale representing the regular blastomeres). For example, an embryo with 7 irregular blastomeres and 20% of fragmentation would be assigned a total sum of 10 embryo score. Cumulative embryo score (CES) was estimated by adding embryo score of each embryo and the mean score of embryo quality (MSEQ) was obtained by dividing the CES with the number of fertilized oocytes. Mean score of embryo transfer (MSET) was calculated by dividing the CES of embryos transferred with the number of transferred embryos.

### 2.4. Assessment of ff o/c-Free DNA Concentration

One mL portion of the total collected follicular fluid specimens was centrifuged at 2000 rpm for 5 min and 0.4 mL cell-free plasma supernatant from each sample was carefully transferred to a new eppendorf tube, boiled for 5 min, quenched on ice, and diluted 5 times with 4 volumes ice cold sterile distilled water.

The concentration of ff o/c-free DNA in each sample was assessed relative to the corresponding concentration of GAPDH PCR product that was determined by conventional quantitative real time PCR-Sybr green detection approach. For this purpose, 5 *μ*L DNA aliquots from each sample, 10 *μ*L commercially available Sybr green master mix containing Sybr Green, Taq DNA polymerase, dNTPs and PCR buffer (Qiagen Inc.), 3 *μ*L GAPDH primer mix [13, 14], and 3 *μ*L sterile distilled water were added to a total volume of 21 *μ*L in an eppendorf tube with light transparent lid and the reactants amplified as previously described [15, 16]. The number of amplification cycles required for initial system recorded GAPDH PCR product identification indicated specimen's sperm plasma GAPDH and ff o/c-free DNA concentration.

Conversion of specimen's number of amplification cycles for initial system recorded GAPDH PCR product identification to genomic DNA concentration (ng/mL) was performed using twenty different clinical whole blood samples to prepare a human DNA stock solution. For this purpose, 200 *μ*L of whole blood aliquots from each sample was processed separately in an automated DNA extraction device and 100 *μ*L from each DNA isolate was combined in 15 mL falcon tube and evaporated to 200 *μ*L by overnight incubation at 70°C with open lid. The purity and DNA concentration (ng/mL) of the resulting human DNA stock solution were determined by UV spectrophotometry [13, 14]. Determination of GAPDH PCR product content of known dilutions of this DNA stock solution as already described was used to generate a standard curve between DNA concentration in ng/mL and amplification cycle. Conversion of follicular fluid specimen's number of amplification cycles to corresponding genomic DNA content (ng/mL) was made with an empirical equation describing this standard curve, the accuracy of which was confirmed manually for three cases at random.

### 2.5. Smoking and Alcohol Assessment

The measurement of smoking status was performed according to the type of the pack-year history. The measurement of alcohol intake was estimated by alcohol units: 1 alcohol unit was considered for 100 mL wine and 200 mL beer, for 30 mL whisky and vodka.

### 2.6. Statistical Analysis

Data were normally distributed. ANOVA was performed for comparison between groups followed by Bonferroni post hoc testing. Bivariate correlation between variables was tested with Pearson's correlation. A *P* value ≤ 0.05 was considered statistically significant. Data are expressed as mean ± standard error of the mean (SEM). SPSS v17 statistical package was used to perform the statistical analysis.

## 3. Results

**Figure 1:**
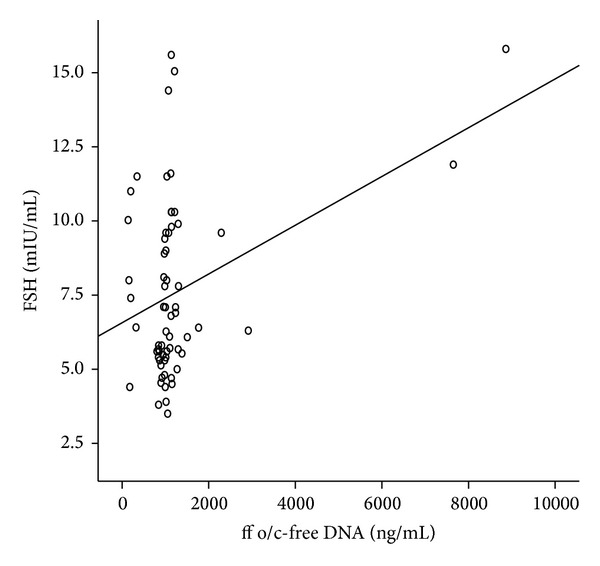
The significant correlation (*r* = 0.37, *P* < 0.05) between basal FSH concentrations and follicular fluid oocyte/cumulus-free DNA levels.

Overall, a positive correlation between ff o/c-free DNA concentrations and basal FSH levels (*r* = 0.37, *P* < 0.05) was found (Figure 1). Except the significant correlation of PCR cycles and the ff o/c-free DNA concentrations (*r* = −0.8, *P* < 0.05), no other correlations were observed between ff o/c-free DNA and the embryological data, in terms of embryo quality and pregnancy.

Table 1 summarizes the parameters studied between the two groups. Besides the significant differences of CES and MSET between the groups, the ff o/c-free concentrations of group 1 were significantly lower compared to group 2. Furthermore, the basic factors (age and BMI) that could have contributed to the number of oocytes, embryo quality, and pregnancy rates were comparable, while the mean basal FSH values, that could have explained the difference in oocyte production, of both groups, were almost similar. Moreover, in group 1 the mean ff o/c-free DNA was significantly correlated with the respective FSH values (*r* = 0.37, *P* < 0.05), the number of selected oocytes (*r* = 0.33, *P* < 0.05), the fertilization rate (*r* = 0.29, *P* < 0.05), and CES (*r* = 0.29, *P* < 0.05). Furthermore, the infertility duration was inversely correlated with cleavage rate (*r* = −0.44, *P* < 0.05), MSEQ (*r* = −0.33, *P* < 0.05), and MSET (*r* = −0.44, *P* < 0.05). On the contrary, in group 2 no correlations were observed between the mean ff o/c-free DNA and the respective parameters examined.

## 4. Discussion

Although it is expected that ff o/c-free DNA, which is released mainly by the oocytes and secondary by the cumulus cells that surrounds the growing oocyte, to be detected in higher levels in cases where more oocytes were retrieved, we showed that increased ff o/c-free DNA levels are associated with limited number of oocytes during oocyte retrieval. The mechanism that possibly explains this phenomenon lays in the fact that ovarian stimulation activates the apoptotic pathway in many recruited oocytes during the process of IVF stimulation. The higher the free DNA in the follicular fluid is, the greater the apoptotic cascade is, which in turn results either in apoptotic oocytes or in limited number of oocytes. The consequence of the above results, through the high free DNA concentrations, seems to impact directly on embryo development and subsequently on embryo quality and implantation potential. The significantly lower embryo quality in group 2 supports the above hypothesis. Moreover, the apoptotic process in granulosa cells through the oxidative stress has been proposed to have a negative effect on IVF outcome, while the higher the apoptotic rate the lower the quality of oocytes [17]. Therefore, cumulus cells of oocytes with low quality had higher apoptotic rates than those with high quality. This assumption is in line with our results where the high free DNA (high apoptotic rate) resulted in limited number of oocytes, yielding relatively poor quality of embryos in terms of MSET in our study.

Although FSH levels were comparable between the two groups, FSH levels of group 2 were not so high to explain the significant difference concerning the number of produced oocytes. Figure 1 depicts the linear positive correlation between overall FSH levels and overall ff o/c-free DNA. This association was found only in group 1 and not in group 2, since in group 1 the number of collected oocytes ranged from 2 to 20. The increase of basal FSH levels (on days 2-3 of the menstrual cycle) is associated with reduction in the number of produced oocytes and, therefore, the increase of ff o/c-free DNA concentration may predict the number and the quality of oocytes during retrieval.

Whether ff o/c-free DNA concentration plays any important role in the IVF outcome remains to be clarified, since so far no relevant data have been reported. Despite the limited number of cases in our study (*n* = 83) some primitive conclusions regarding this biomolecular parameter can be drawn. So far, it has been reported that increased serum plasma cell-free DNA was associated with significantly low pregnancy rates in women undergoing IVF-ET treatments [9]. Besides the limited number of cases in that study (*n* = 37), another limitation is that cell-free DNA was studied in serum and not in the follicular fluid, which is the ambient environment of the oocytes. Therefore, the estimation of ff o/c-free DNA may directly link the free DNA with oocytes, which are the main gametes responsible for early embryo development, implantation potential, and partially for the IVF outcome. In the present study, although a higher number of cases were studied compared to the study of Czamanski-Cohen et al. [9] (*n* = 83 versus *n* = 37), the percentages of either positive hCG or clinical pregnancy were comparable between the two groups. The same comparable results were obtained even after the comparison of ff o/c-free DNA concentrations between pregnant and not pregnant women, indirectly supporting the possibility that IVF conception does not depend upon this biomolecular marker. This suggestion was also supported after the performance of binary logistic regression analysis using pregnancy as a dependent variable and ff o/c-free DNA concentrations as covariate (*P* > 0.05).

The paradox that arises regarding the pregnancy rate in the two groups is firstly why group 1 has a relative unexpected low pregnancy rate given the high number of collected oocytes and secondly why the group 2 demonstrated a rather high pregnancy rate given the 1 oocyte/retrieval. Do ff o/c-free DNA concentrations play any role in this phenomenon? It appears, according to the above observations, that the high number of produced oocytes in group 1 does not correspond to equivalent quality of oocytes. In other words, the large number of collected oocytes in group 1 is not in line with excellent quality oocytes. It seems that ff o/c-free DNA concentrations may exert a negative effect in the quality of produced oocytes in such a way that initiation of apoptosis is not apparent. Moreover, the process of apoptosis initiates before the morphological signs are visible [18]; therefore an atretic follicle may not be easily characterized/seen because not all cumulus cells have undergone cell death. Regarding the relative high pregnancy rate in group 2, the role of ff o/c-free DNA concentration is more apparent since the high levels of ff o/c-free DNA appear to block the production of spare oocytes, limited to one, which according to stimulation and biological rationale appears to favor the quality of oocytes as more suitable for fertilization and development.

Conclusively, we recorded herein for the first time free DNA in the follicular fluid, an apoptotic marker of the ambient environment of the female gametes, as a quality parameter of oocytes, embryos, and IVF outcome. Evaluation of this new marker may eventually enhance the success outcome of ART couples. More studies are required to clarify whether ff o/c-free DNA can be used as a marker of embryo quality and IVF result.

## Figures and Tables

**Table 1:** Characteristics (mean ± SEM) of women according to the number of oocyte/cumulus complexes (CoCs) collected during ovum pickup.

CoCs	≥2	=1	*P* value
Number of cases	63	21	
Age of woman (y)	35.8 ± 0.6	37.47 ± 1.1	NS
BMI of woman (kg/m^2^)	25.93 ± 0.6	25.54 ± 1.2	NS
Basal FSH (mIU/mL)^1^	7.67 ± 0.4	7.51 ± 0.7	NS
Infertility duration (y)	3.46 ± 0.2	3.44 ± 0.5	NS
Basal LH (mIU/mL)^1^	4.63 ± 0.3	4.61 ± 0.6	NS
Estradiol (pg/mL)^2^	1722.3 ± 128.18	1735 ± 262.3	NS
CoCs	7.23 ± 0.5	1	**<0.05**
FR (%)	67.37 ± 2.7	76.19 ± 5.5	NS
CR (%)	82.25 ± 3.7	80.95 ± 8.7	NS
CES	32.07 ± 2.9	6.91 ± 0.9	**<0.05**
MSEQ	8.48 ± 0.4	6.91 ± 0.9	NS
MSET	9.69 ± 0.4	6.91 ± 0.9	**<0.05**
Follicular fluid oocytes/cumulus-free DNA (ng/mL)	1165.65 ± 142.7	2077.31 ± 629.9	**<0.05**
Smoking status (packs-year history)	0.21 ± 0.0	0.14 ± 0.0	NS
Alcohol status (alcohol units)	0.61 ± 0.1	0.33 ± 0.2	NS
Positive hCG (%)	28.6%	14.3%	NS
Clinical pregnancy (%)	23.8%	14.3%	NS

^1^FSH and LH values on day 2 of the menstrual cycle.

^2^Estradiol values on the day of human chorionic gonadotropin (hCG).

y, years; BMI, body mass index; FR, fertilization rate; CR, cleavage rate; CES, cumulative embryo score; MSEQ, mean score of embryo quality; MSET, mean score of embryo transfer.
